# Supplementary material for: Observed Antarctic sea ice expansion reproduced in a climate model after correcting biases in sea ice drift velocity
Source: Nat Commun. 2021 Feb 16;12:1060. doi: 10.1038/s41467-021-21412-z (PMC7887216; doi:10.1038/s41467-021-21412-z)
Supplement: Supplementary file 1 — Supplementary Information [file 41467_2021_21412_MOESM1_ESM.pdf]

Supporting Information for "Observed Antarctic sea ice expansion reproduced in a climate model after correcting biases in sea ice drift velocity"

Shantong Sun[1,2] and Ian Eisenman[1]

[1]Scripps Institution of Oceanography, University of California San Diego, USA
[2]California Institute of Technology, USA

Budget analysis: dynamic versus thermodynamic sea ice processes

In order to aid in the interpretation of the influence of sea ice motion on sea ice area in the CESM simulations, we separate the sea ice concentration changes due to dynamic processes (ice transport and ridging) from those due to thermodynamic processes (melting and freezing) according to

$$\frac{\partial C}{\partial t} = \mathcal{T}_{\text{dyn}} + \mathcal{T}_{\text{therm}}, \tag{S1}$$

where C is the sea ice concentration, $\mathcal{T}_{\text{dyn}}$ represents the ice concentration tendency due to dynamic processes, and $\mathcal{T}_{\text{therm}}$ represents the ice concentration tendency due to thermodynamic processes. The two tendency terms are diagnosed in the model and their monthly-mean values are reported in the model output.

Integrating Equation (S1) in time, we separate the sea ice concentration at time t into two parts,

$$C = \int_0^t \frac{\partial C}{\partial t'} dt' = \int_0^t \mathcal{T}_{\text{dyn}} dt' + \int_0^t \mathcal{T}_{\text{therm}} dt'. \tag{S2}$$

This allows us to decompose the trend in sea ice concentration into separate parts representing dynamic and thermodynamic contributions:

$$s = s_{\text{dyn}} + s_{\text{therm}}, \tag{S3}$$

where s represents the long-term linear trend in sea ice concentration, s_{dyn} represents the trend in the dynamic contribution $\int_0^t \mathcal{T}_{\text{dyn}} dt'$, and s_{dyn} represents the trend in the thermodynamic contribution $\int_0^t \mathcal{T}_{\text{therm}} dt'$.

With this framework, differences in the sea ice concentration trend between two CESM simulations can be attributed to contributions from dynamic and thermodynamic processes:

$$\delta s = \delta s_{\text{dyn}} + \delta s_{\text{them}}. \tag{S4}$$

Next, we integrate Equation (S4) over latitude in the Southern Hemisphere. Considering the linear trend in the annual-mean meridionally-integrated sea ice area, which is plotted in Figure 3 of the main text, this budget analysis allows us to separate the dynamical and thermodynamic contributions to the difference between each LENS run and ObsVi run. The results of this analysis are plotted in Supplementary Figure 9.

The contributions due to dynamic processes and thermodynamics processes largely cancel (Supplementary Figure 9). Changes in the sea ice area trend are approximately consistent with a larger northward sea ice transport in the Ross Sea and the Weddell Sea in all of the ObsVi runs than in the corresponding LENS runs. In the Indian Ocean sector, by contrast, the budget analysis indicates decreased northward sea ice transport in the ObsVi runs compared with the LENS runs. Note, however, that stronger northward sea ice export in these simulations does not always correspond with expanded sea ice cover (Supplementary Figure 8).

Sensitivity of simulations to spinup conditions

The difference in spin up behavior between the ObsVi runs and the ERAWind runs raises the possibility that the results during the 1992-2015 analysis period may be sensitive to the choice of spin up conditions. We tested this by carrying out several additional sets of simulations.

First, since the ERAWind runs are forced during spin up by repeating a single year of the observations whereas the ObsVi runs are forced by repeating the 1992-2015 mean annual cycle, we carried out three runs that are identical to ObsVi except that they are spun up during 1960-1991 using the 1992 observed ice motion

field each year (referred to as ObsVi_1992Spinup). The decline during the first part of the spin up period is somewhat larger on average in the ObsVi_1992Spinup runs than in the ObsVi runs, and the ObsVi_1992Spinup runs appear to take longer to stabilize during the spin up period (red lines in Supplementary Figure 11a). The sea ice area trends during 1992-2015 in two of the ObsVi_1992Spinup runs fall within the spread of the three ObsVi runs, but one of the ObsVi_1992Spinup runs has sea ice retreat (Supplementary Table 1). This may be related to the ObsVi_1992Spinup runs possibly not being sufficiently spun up, although some of the differences between the ObsVi and ObsVi_1992Spinup runs may simply be due to internal variability, given the limited number of runs in each ensemble.

As a second test of the sensitivity to the choice of spin up conditions, we carried out three runs that are identical to ERAWind except that they are spun up during 1960-1991 using the 1992 forcing in each year (referred to as ERAWind_1992Spinup), similar to the ObsVi_1992Spinup runs. The ERAWind_1992Spinup runs behave fairly similarly to the ERAWind runs throughout the 1960-2015 simulation period (red lines in Supplementary Figure 11b). The sea ice area trends during 1992-2015 in two of the ERAWind_1992Spinup runs fall within the spread of the three ERAWind runs, but one of the ERAWind_1992Spinup runs has sea ice expansion (Supplementary Table 1). As with the ObsVi_1992Spinup runs, it is difficult here to separate differences due to spin up conditions from the effects of internal variability.

As a third test of the sensitivity to the choice of spin up conditions, we carried out three runs that are identical to ERAWind except that they are spun up during 1960-1991 using the 1992-2015 mean annual cycle in surface winds (refereed to as ERAWind_ClimSpinup). These runs behave markedly differently, with the ice area remaining well above the LENS runs during the entire simulation period and a relatively abrupt decline in sea ice area occurring during 1995-2000 (green lines in Supplementary Figure 11b). This leads to 1992-2015 sea ice decline that is faster than the LENS runs (Supplementary Table 1). The behavior of the ERAWind_ClimSpinup runs may be related to issues associated with the smoothness of the climatological forcing compared with a typical year which has more short-term variability. By contrast, this issue does not appear to be substantially influencing the ObsVi runs: the ObsVi_1992Spinup runs behave fairly similarly to the ObsVi runs, whereas the ERAWind_1992Spinup runs do not behave similarly to the ERAWind_ClimSpinup runs.

Lastly, in order to test the long-term influence of using specified ice motion, we carried out three additional runs in which the sea ice motion is specified to follow the observed 1992-2015 mean annual cycle each year (referred to as Ob-

sVi_ClimThroughout), as well as three runs with the ice motion specified to follow the observed 1992 field each year (referred to as ObsVi_1992Throughout). These runs are identical during 1960-1991 to the ObsVi and ObsVi_1992Spinup runs, respectively. One of the ObsVi_1992Throughout runs has ice retreat and two have ice expansion (Supplementary Table 1), which may be related to the recovery from the low in 1980 during the spin up period (red lines in Supplementary Figure 11c). This suggests that the ObsVi_1992Spinup runs may not be fully spun up in 1992, as noted above. In the ObsVi_ClimThroughout runs, which appear to spin up more quickly during the 1960-1991 spin up period (blue lines in Supplementary Figure 11c), the sea ice retreats in all three runs at a rate similar to the LENS runs (Supplementary Table 1). This suggests that the Antarctic sea ice expansion in the main runs (ObsVi) occurs due to the changes in the sea ice drift velocity during recent decades, rather than simply being an artifact of the model adjusting to specified ice motion.

In addition to apparent spin up issues in the ObsVi_1992Spinup runs, a shortcoming of the ObsVi_1992Spinup and ERAWind_1992Spinup supplemental runs described in this section is that they may become artificially equilibrated to the forcing in the first year of the 1992-2015 analysis period. This is in contrast to the main simulations: the ObsVi runs have an average forcing during the spin up period, and the ERAWind runs have evolving forcing during the last 13 years of the spin up period (1979-1991).

Name	SH trend	SH corr	NH trend	NH corr
Observations	32.7		-64.8	
LENS-2	-49.2	0.08	-67.1	-0.06
LENS-4	-31.2	-0.13	-26.9	0.11
LENS-6	-29.1	-0.12	-54.0	-0.29
ObsVi-2	15.2	-0.06	-16.7	-0.15
ObsVi-4	29.6	-0.34	-11.3	-0.27
ObsVi-6	3.8	-0.13	-2.7	-0.40
ERAWind-2	-13.1	0.38	-25.0	-0.35
ERAWind-4	-6.8	0.62	-40.6	0.57
ERAWind-6	-0.2	0.59	-34.1	-0.36
ObsVi_1992Spinup-2	15.7	-0.28	-26.5	-0.26
ObsVi_1992Spinup-4	-31.8	0.37	-66.8	-0.16
ObsVi_1992Spinup-6	9.2	-0.13	-56.6	-0.27
ERAWind_1992Spinup-2	-0.6	0.30	-32.7	0.29
ERAWind_1992Spinup-4	14.0	0.64	-20.5	0.34
ERAWind_1992Spinup-6	-13.7	0.35	-48.5	-0.19
ERAWind_ClimSpinup-2	-80.2	0.25	4.2	-0.07
ERAWind_ClimSpinup-4	-72.1	0.37	-35.3	0.01
ERAWind_ClimSpinup-6	-81.2	0.49	-27.3	0.13
ObsVi_ClimThroughout-2	-30.5	0.24	-63.1	-0.05
ObsVi_ClimThroughout-4	-24.4	-0.18	-42.4	0.07
ObsVi_ClimThroughout-6	-12.8	-0.12	-45.0	-0.18
ObsVi_1992Throughout-2	4.4	-0.25	-37.7	0.03
ObsVi_1992Throughout-4	-21.0	-0.01	-32.9	0.33
ObsVi_1992Throughout-6	13.0	-0.38	-41.9	0.03

Table 1: Linear trend in annual-mean ice area during 1992-2015 in each hemisphere ("trend", in units of 10^3 km^2/yr) for observations, main simulations, and supplemental simulations. A measure of the agreement with observed year-to-year changes is also included ("corr"), which is calculated as the linear correlation coefficient r with observations of the detrended annual-mean ice area during 1992-2015.

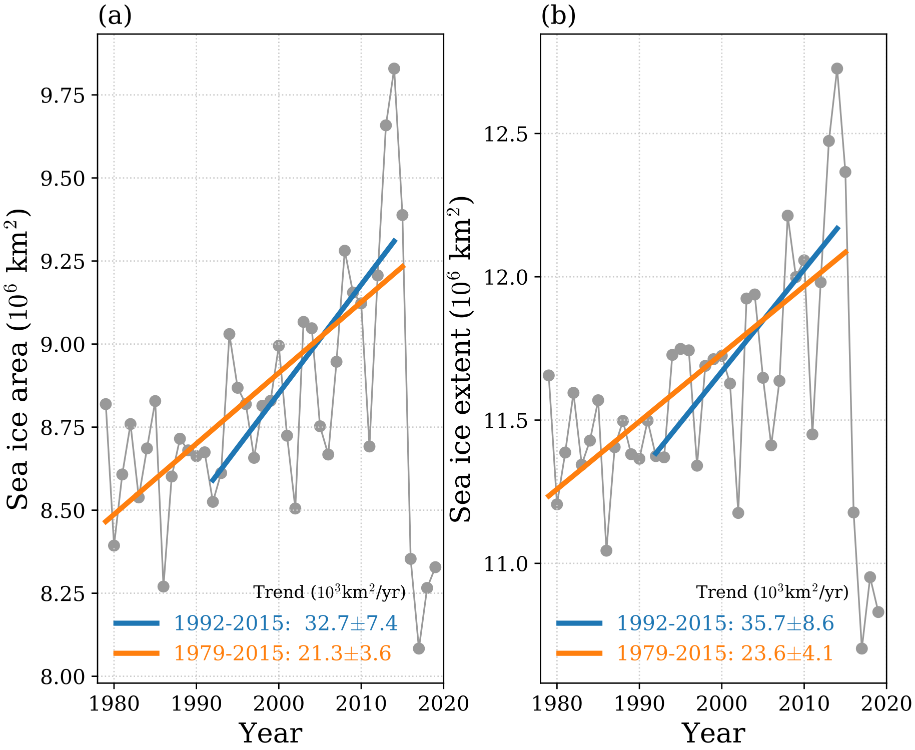

Supplementary Figure 1: Satellite-derived observations of Antarctic sea ice cover during 1979 to 2019. (a) Annual-mean sea ice area. This study focuses on ice area, but ice extent (shown in panel b) is also often considered. (b) Annual-mean sea ice extent. In both panels, the linear trends during 1979-2015 (orange straight line) and 1992-2015 (blue straight line) are indicated. For comparison, the linear trend in the Arctic sea ice area and sea ice extent during 1979-2015 are -64.8×10^3 km^2/yr and -68.9×10^3 km^2/yr, respectively.

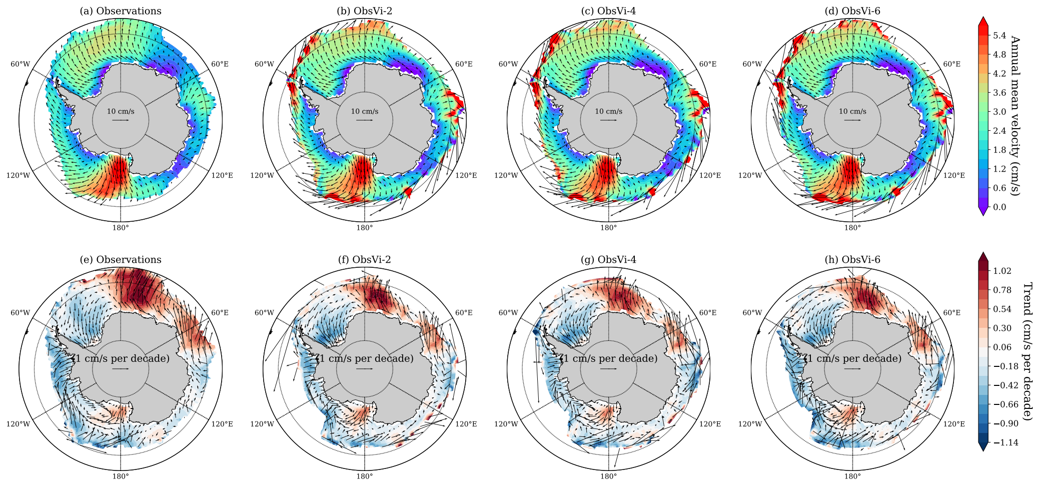

Supplementary Figure 2: Sea ice drift velocities in the observations and ObsVi runs. The top row shows the 1992-2015 mean value of the drift velocity, and the bottom row shows the 1992-2015 trend in annual-mean drift velocity. In all panels, the shading indicates the meridional component of the velocity or velocity trend. Note the agreement between the three ObsVi runs and the observations, as expected based on the simulation setup.

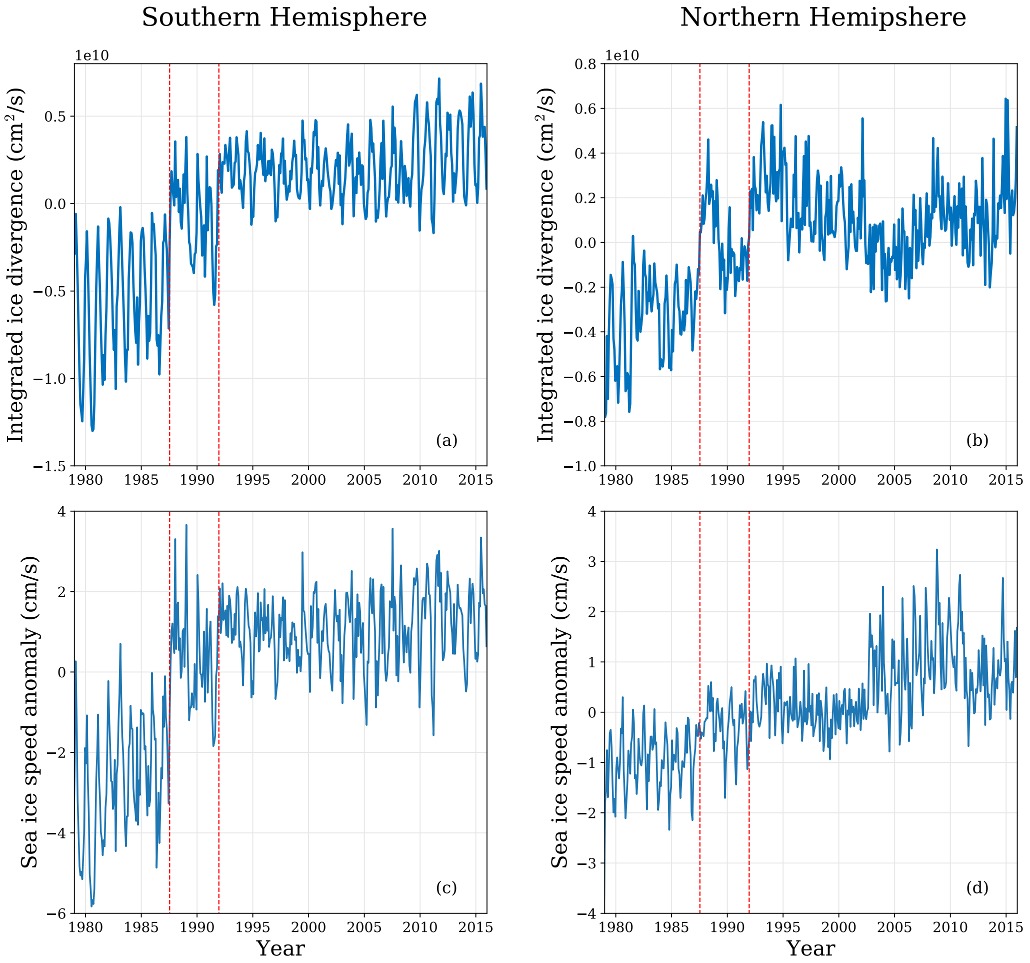

Supplementary Figure 3: Issue with observational estimate of sea ice motion before 1992. The area-integrated sea ice velocity divergence (top) and area-averaged sea ice speed anomaly (bottom) are plotted for the Southern Hemisphere (left) and the Northern Hemisphere (right). The sea ice speed anomaly is calculated related to the long-term mean during 1979-2015. The transition from the Scanning Multichannel Microwave Radiometer (SMMR) to the Special Sensor Microwave/Imager (SSM/I) on July 9, 1987, and the transition from the SSM/I sensor flown on the Defense Meteorological Satellite Program (DMSP) F8 satellite to the SSM/I sensor flown on the DMSP F11 satellite on December 3, 1991, are marked with red dashed lines on each plot. Note the jumps in the ice drift data associated with these sensor transitions.

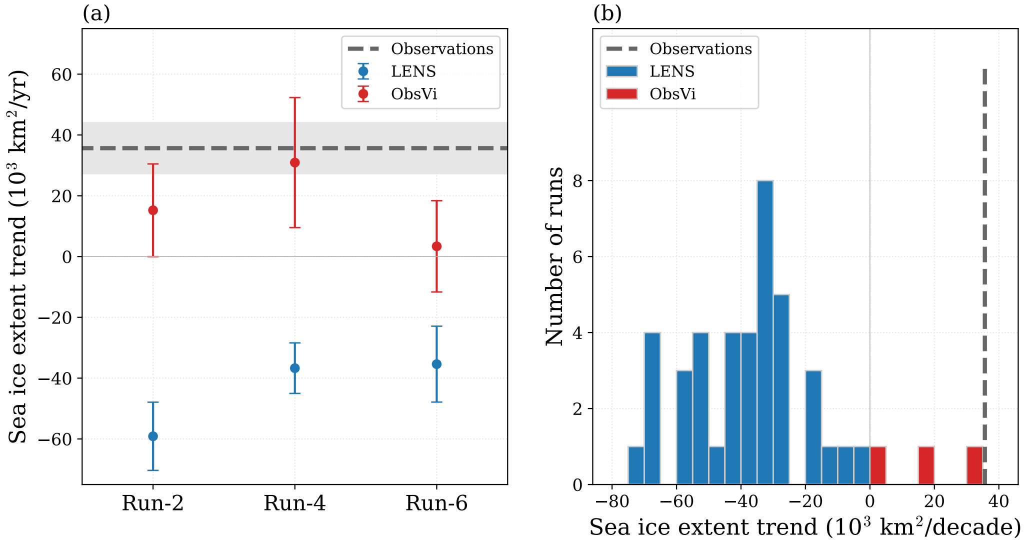

Supplementary Figure 4: As in Figure 2 in the main text, but using ice extent rather than ice area.

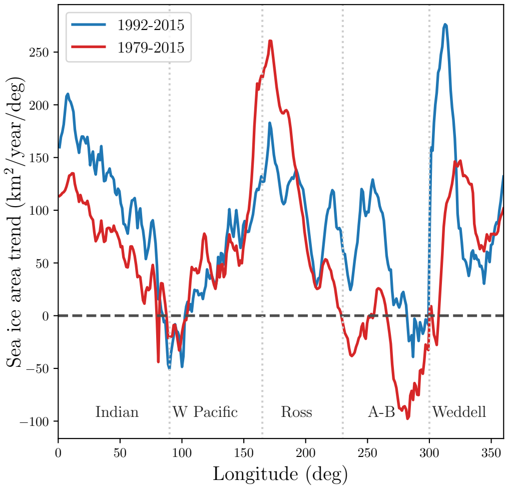

Supplementary Figure 5: Linear trend in the observed annual-mean meridionally-integrated sea ice area. Values calculated during 1992-2015 (blue) are compared with values calculated during 1979-2015 (red).

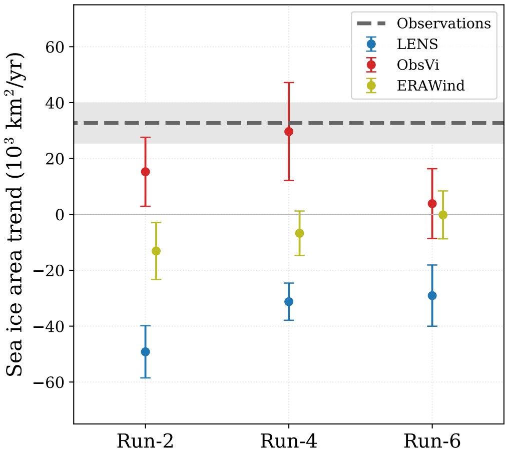

Supplementary Figure 6: As in Figure 2a in the main text, but also including the ERAWind runs. Note that the ERAWind runs are offset slightly to the right to avoid overlap.

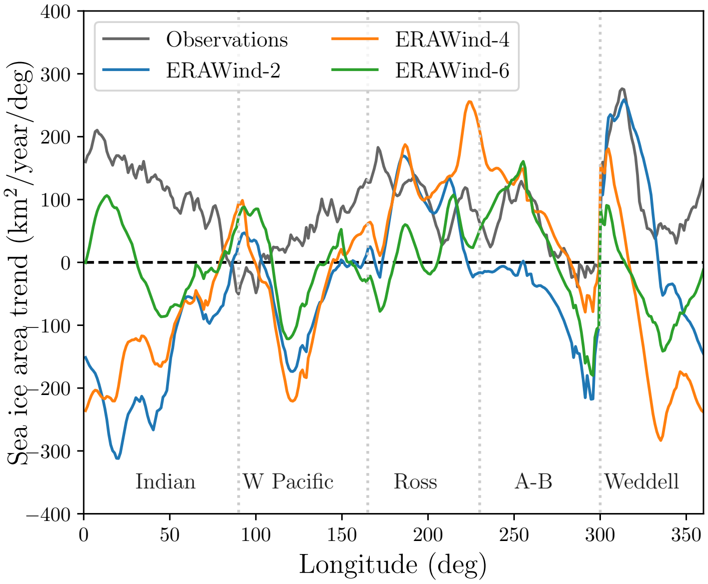

Supplementary Figure 7: As in Figure 3 in the main text, but for the ERAWind runs.

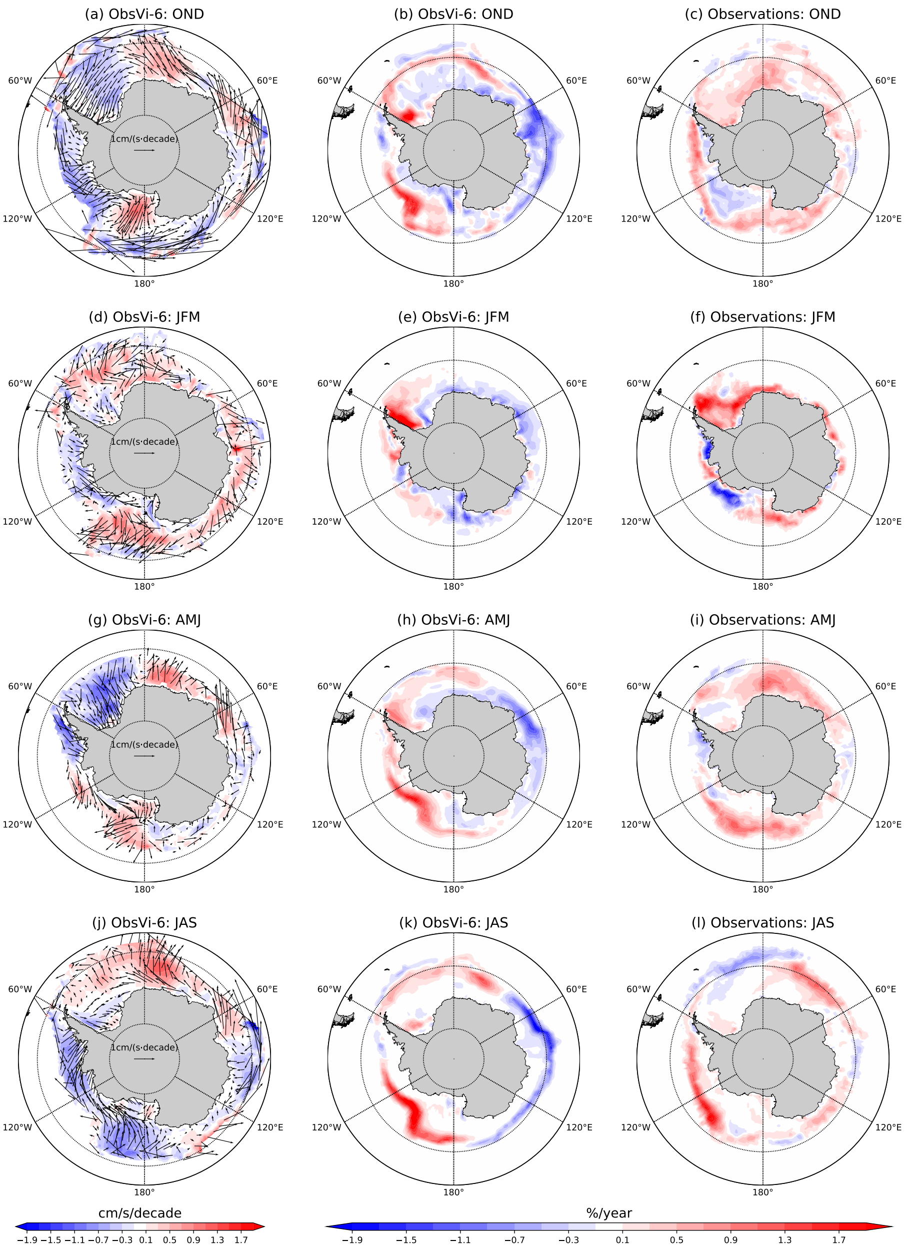

Supplementary Figure 8: Relationship between trends in ice velocity and trends in ice concentration in the ObsVi runs and observations. Each row represents a different season. The columns represent (left) the linear trend in seasonal-mean sea ice velocity, (center) the linear trend in the seasonal-mean sea ice concentration in the ObsVi-6 run, and (right) the linear trend in the seasonal-mean sea ice concentration in the observations. All trends are computed during 1992-2015.

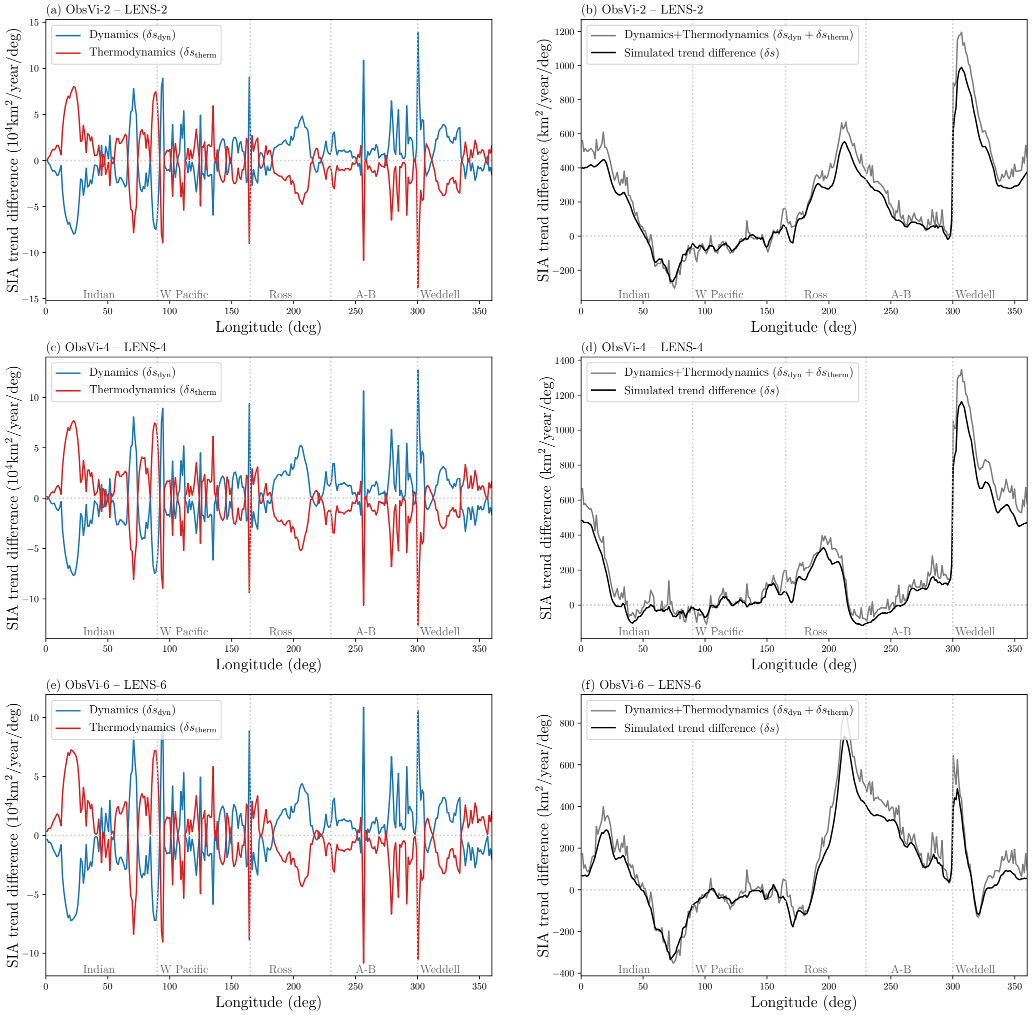

Supplementary Figure 9: Results of the dynamic vs thermodynamic budget analysis of the sea ice area trend. The rows represent the difference between (top) ObsVi-2 and LENS-2, (middle) ObsVi-4 and LENS-4, and (bottom) ObsVi-6 and LENS-6. The columns represent (left) the contributions to the sea ice area trend difference due to dynamic processes (blue) and thermodynamic processes (red), and (right) the sum of the two terms plotted in the left column (gray) compared with the actual difference in the total trend (black) as a test of the accuracy of the budget analysis. Note that the relatively small difference between the gray line and the black line is expected to be due to the usage of monthly-mean model output in the budget analysis.

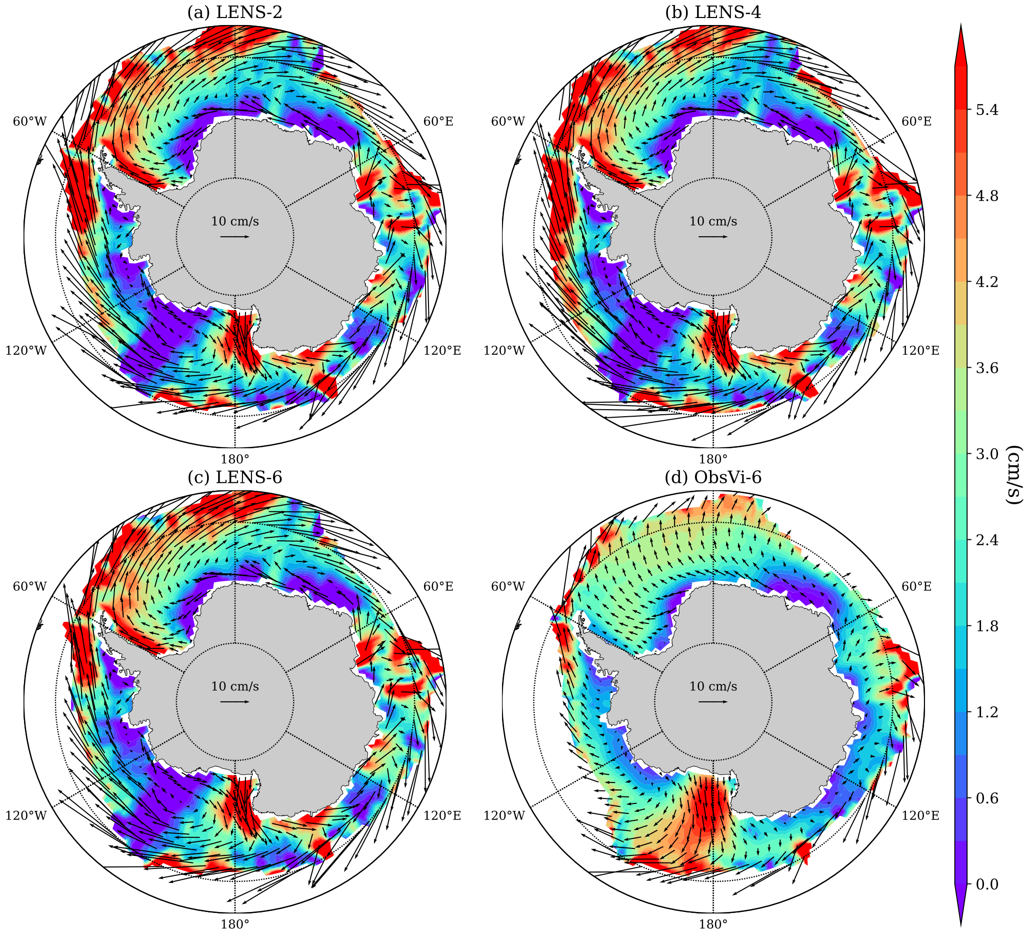

Supplementary Figure 10: 1992-2015 mean value of the drift velocity in LENS runs and ObsVi-6. Shading indicates the meridional component of the velocity. Note that the annual-mean sea ice drift velocities in the observations, ObsVi-2, and ObsVi-4 are approximately the equivalent to ObsVi-6 (Supplementary Figure 2). The LENS runs show mainly eastward movement of sea ice, whereas in ObsVi-6 the sea ice movement is mainly in the meridional direction.

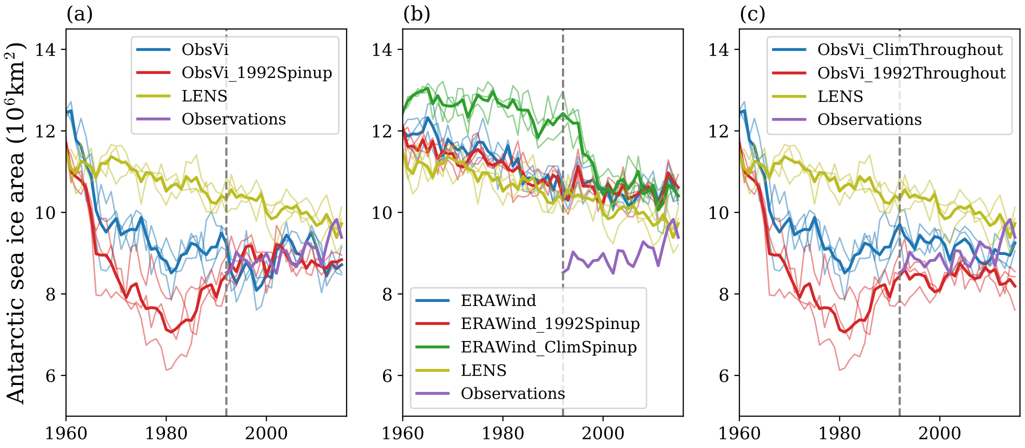

Supplementary Figure 11: Annual-mean Antarctic sea ice area evolution during the entire simulations, including the spinup period. The thin lines represent each of the ensemble members and the thick lines indicate the ensemble-mean of each 3-member ensemble. The LENS runs and observations are repeated in each panel for comparison. The gray dashed lines indicate the year 1992.

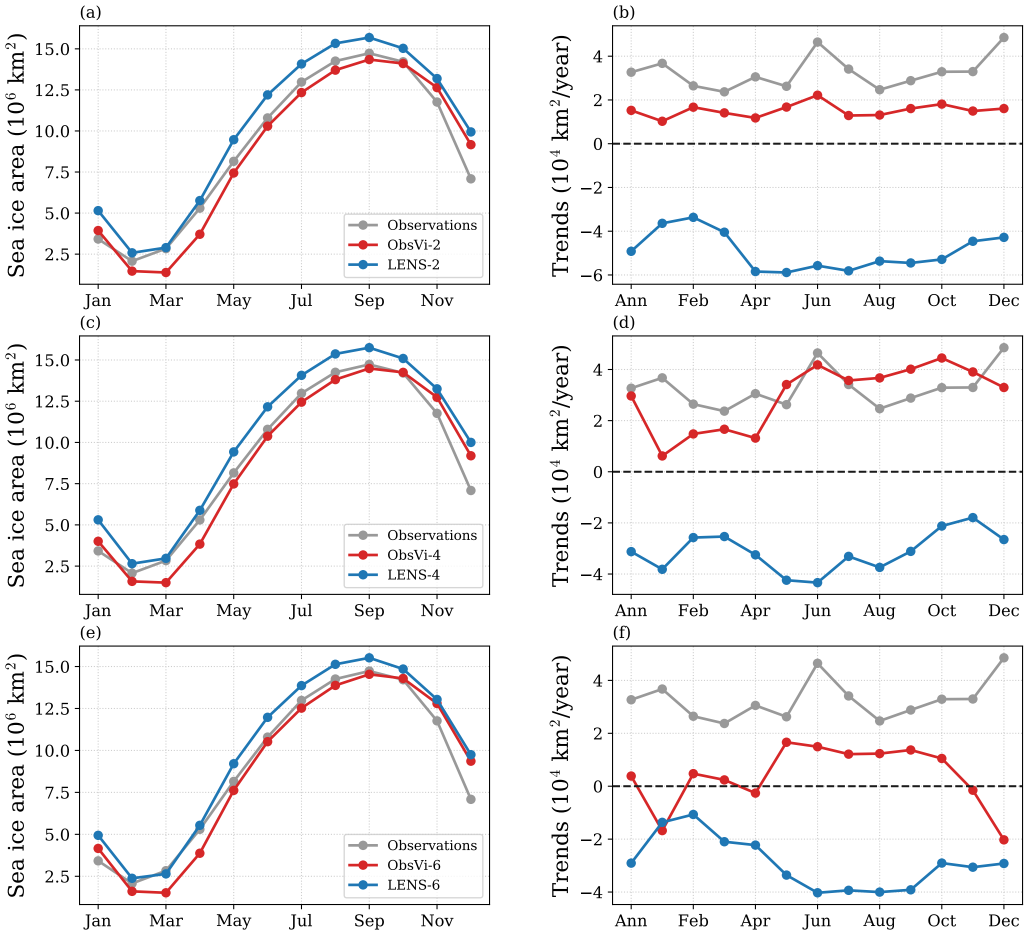

Supplementary Figure 12: Seasonal cycle of the mean state and the linear trend in Antarctic sea ice area during 1992-2015. Observations are repeated in each panel as a gray line. In the right panels, "Ann" represents the linear trend in the annual-mean sea ice area. Note that here each panel shows all simulations with a given index, rather than showing a single set of simulations.
